# Effect of Filter Types on Physicochemical Properties, Volatile Compounds, and Sensory Evaluations of Purified Water by Point-of-Use Water Treatment

**DOI:** 10.3390/foods10081958

**Published:** 2021-08-22

**Authors:** Mi-Ran Kim, JeongAe Heo, Sang Sook Kim, Eui-Cheol Shin, Chang Guk Boo, Han Sub Kwak

**Affiliations:** 1 Research Group of Food Processing, Korea Food Research Institute, Wanju-gun 55465, Korea; ranni1027@kfri.re.kr (M.-R.K.); sskim@kfri.re.kr (S.S.K.); 2 Technical Assistance Center, Korea Food Research Institute, Wanju-gun 55465, Korea; heo.jeongae@kfri.re.kr; 3 Department of Food Science, Gyeongsang National University, Jinju-si 52725, Korea; eshin@gnu.ac.kr (E.-C.S.); dbs7987@naver.com (C.G.B.); 4 KFRI School, University of Science and Technology, Wanju-gun 55465, Korea

**Keywords:** point-of-use water treatment, purified water, volatile organic compounds, sensory discrimination, consumer acceptance

## Abstract

This study investigated purified water from four different filter types for removing minerals, anions, and volatile organic compounds (VOCs), and affecting sensory perception and consumer acceptability. Ultrafiltration (UF), CSM-ultrafiltration (CU), alumina nanofiber (AN), and reverse osmosis (RO) filters were used for a point-of-use water treatment system with a pre-carbon filter (PR) and post-carbon filter (PO). Filters efficiently removed VOCs, which could negatively affect the sensory perception of water. The total VOC concentration of tap water (TW) (14.97 µg/Kg) was reduced by 70% by the PR, 75.3–88.7% by the PR-main filter, and >97% by the PR-RO-PR. Using the polarized sensory position test, the subjects clearly discriminated TW from the samples; however, most of the purified water was not. The difference in the mean ratings of consumer acceptability among the purified samples was <1 except for PR-RO-PO in consumer testing. These results suggested that although there are differences in the capability of different filter types to eliminate minerals, anions, and VOCs, overall consumers did not identify sensory differences among them, and demonstrated similar consumer acceptability of the purified water produced. Simply applying a pre-carbon filter for TW treatment is enough to minimize VOCs, which negatively influence consumer acceptability.

## 1. Introduction

Tap water (TW), which is extracted from a lake or river, moved to a filtration plant to make purified water, and transferred to homes through pipes, is essential to households for cooking and drinking. Although a large proportion of people directly use TW for drinking, many people do not drink it. Instead, people purchase bottled water from stores or set up a water purifier at home for drinking water. The main reasons for not drinking TW are related to the organoleptic issue generated during the filtration process [1,2], safety concerns [3,4], unreliable small water systems [5], and low quality in many underdeveloped countries.

An organoleptic issue with drinking TW is that the disinfectants used in filtration plants react with volatile organic compounds (VOCs; [6]). VOCs in TW are one of the main reasons for not drinking TW directly [7]. In Korea, 49% of Seoul residents drink tap water; however, 84% of them drink tap water after boiling it [8]. Ikem [9] showed that bottled water had a lower amount of total VOCs and total trihalomethanes than TW abstracted from the Missouri River in the USA. According to Doria [1], many Canadian and French residents drink bottled water instead of TW for organoleptic reasons. Some studies have shown that consumers drink bottled water for safety issues, while other consumers drink purified TW to remove off-flavor issues in TW [4,10].

Studies on drinking bottled water versus TW have been conducted by several researchers; however, there is a lack of studies on purified TW, especially on the organoleptic aspect, compared to the safety issue [5]. Point-of-use (POU) water treatment is used in developed and developing countries, primarily in Asia, North America, and Europe, because of its lower cost, and ease of installation and maintenance [11]. Using a POU water treatment is a way to supply high-quality water by filtering TW immediately prior to drinking water at home, and is more environmentally friendly than drinking bottled water due to the reduced consumption of plastic. The effects of POU water treatment at home are presented from various aspects. Stalter et al. [12] showed POU treatment of TW removed up to 88% of fluoride and up to 89% of bacteria. Serpieri et al. [13] showed that a carbon filter followed by ultraviolet sterilization removed VOCs such as trichloromethane, trichloroethene, and benzene compounds in TW by up to 90%. The heavy metals in TW were partially removed by a home water purifier in Qatar [14], and POU water treatment using anionic silver nanoparticles on cellulose filter paper supplied microbially safe drinking water [15].

Several types of filters have been applied in POU water treatment. The two most widely used filter types are reverse osmosis (RO) and ultrafiltration (UF). RO membranes have extremely small pore sizes to prevent microorganisms and obtain pure water; however, their filtration efficiency is low [16]. UF is known for its efficiency and low filtration cost; however, it cannot filter all microorganisms due to the larger pore size than RO [17]. Other types of filters are also used in POU water treatment. CSM-UF (CU) is a spiral form of the UF membrane with a smaller pore size than UF and a higher filtration efficiency compared to the RO (www.csmfilter.com accessed on 17 August 2021). An alumina nanofiber filter (AN) is composed of small fibers made from aluminum metal or aluminum-coated materials [18]. Although some studies have presented the effect of POU water treatment, comparisons across the primary filter types and filtration steps in removing volatile compounds and the discrimination of purified water via sensory evaluations have not been fully presented. Therefore, the objectives of this study were to investigate the effect of four different main filter types (UF, CU, RO, and AN) and a post-carbon filter (PO) on removing minerals, anions, and VOCs; discriminating purified water using sensory evaluation techniques; and measuring consumer acceptance of purified water.

## 2. Materials and Methods

### 2.1. Water Filtration System

**Figure 1:**
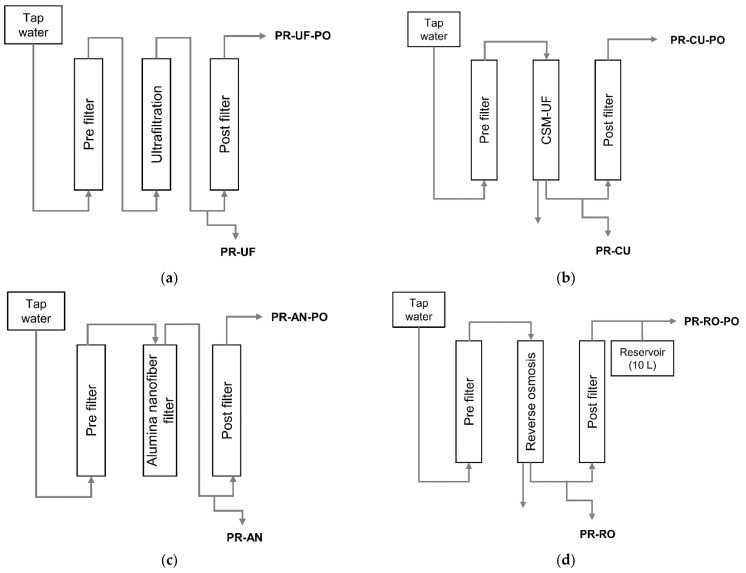
The point-of-use water treatment system used in this study: (**a**) ultrafiltration (UF); (**b**) CSM-ultrafiltration (CU); (**c**) alumina nanofiber filter (AN); and (**d**) reverse osmosis filter (RO). PR and PR mean pre-carbon and post-carbon filter, respectively.

The four POU water treatments are shown in Figure 1. Four main filters (UF, CU, RO, and AN) were used in the experiment. Each main filter was composed of a pre-carbon block filter (PR) and was coded as PR-UF, PR-CU, PR-RO, or PR-AN. A PO was also added to these filters and coded as PR-UF-PO, PR-CU, PR-RO-PO, or PR-AN-PO to investigate the effect of the post-carbon filter on water quality. Filters were purchased from an online filter store (Filter114 Co., Seoul, Korea). The UF, CU, RO, and AN filters were produced by Kolon Industry, Co. Ltd. (Seoul, Korea), Toray Advanced Materials Korea Inc. (Seoul, Korea), Woongjin Chemical Co. Ltd. (Seoul, Korea), and Argonide Co. (Sanford, FL, USA), respectively. Approximately 500 L of tap water was passed through the filters prior to the experiment. The TW used in this study was extracted from Yongdam Lake (Jinan-gun, Korea, geological coordinates 35.9457, 127.5248), purified in Gosan filtration plant (Wanju-gun, Korea, geological coordinates 35.9779, 127.2271), and drained at the Korea Food Research Institute (Wanju-gun, Korea, geological coordinates 35.8352, 127.0496).

### 2.2. Mineral and Anion Analyses of Purified Water

The minerals in the purified water (Ca, Na, K, and Mg) were analyzed according to the Korean Food Standard Codex [19]. Purified water (1 g) was placed in a microwave-safe Teflon vessel (Cowie Technology, Middlesbrough, UK). Then, 8 mL of HNO_3_ (electronic grade, Dongwoo Fine Chemical Co. Ltd., Seoul, Korea) and 2 mL of H_2_O_2_ (electronic grade, Dongwoo Fine Chemical Co. Ltd.) were added to each sample. After placing the vessels in a microwave digestion system (Multiwave ECO, Anton Paar, Graz, Austria), the samples were heated at 100 °C for 10 min, digested for 2 min, heated again at 180 °C for 10 min, and digested again for 30 min. After the final digestion, the samples were slowly cooled to 70 °C. They were then transferred into a measuring cylinder, which was subsequently filled with deionized water to 50 g, and the contents were filtered through filter paper (Whatman No. 41, Maidstone, UK). Ca, Na, K, and Mg standard solutions (1000 mg/L; AccuStandard, New Haven, CT, USA) were each diluted to concentrations of 1, 5, 25, and 100 mg/Kg with 2% HNO_3_ solution. Inductively coupled plasma atomic emission spectroscopy (ICP-AES; Avio 500, Perkin Elmer Inc., Waltham, MA, USA) analysis was performed at 589.592 and 279.079 nm for Na and Mg, respectively.

The anions in the purified water (Cl, NO_3_, SO_4_) were analyzed according to US EPA 300.1 protocol [20] using an ion chromatograph (IC; ICS-1500 model, Dionex, Sunnyvale, CA, USA) at the Korea Institute of Geoscience and Mineral Resources (Daejeon, Korea).

### 2.3. Identification of Volatile Compounds of Purified Water by GC-MS

A solid-phase microextraction (SPME) fiber coated with 75 μm carboxen/polydimethylsiloxane (CAR/PDMS) (needle size: 24 Ga) (Supelco Co., Bellefonte, PA, USA) was used to collect the volatile compounds. According to [21], the CAR/PDMS fiber generates the most appropriate information in the aqueous matrix. The sample (100 mL) was placed in a bottle and sealed using an aluminum cap. The sample was then heated in an 80 °C heating block for 20 min, and the volatile compounds were collected for 20 min in the SPME fiber. The collected volatile compounds were analyzed using gas chromatography-mass spectrometry (GC-MS, Agilent 7890A and 5975C, Agilent Technologies, Santa Clara, CA, USA) with an HP-5MS column (30 m × 0.25 mm i.d. × 0.25 µm film thickness). The oven temperature was maintained at 40 °C for 5 min and then increased to 200 °C at a rate of 5 °C/min. The injector temperature was set to 220 °C, helium carrier gas flowed in at 1.0 mL/min, and the split ratio was 1:10. Compounds separated from the total ionization chromatogram (TIC) were identified using the mass spectrum library (NIST 12), ion fragmentation pattern, and a reference [22]. Volatile compounds were determined using a semiquantitative method based on conversion into peak areas of pentadecane (0.005 μg) as a standard and expressed as μg/1 Kg. The retention index (RI) was calculated using Equation (1):

RIx = 100n + 100 ((tRx − tRn)/ (tRn + 1 − tRn))(1)

where RIx is the RI of the unknown compound, tRx is the retention time of the unknown compound, tRn is the retention time of the n-alkane, and tRn + 1 is the retention time of the next n-alkane. tRx is between tRn and tRn + 1 (n = number of carbon atoms).

### 2.4. Polarized Sensory Positioning Test of Purified Tap Water

The polarized sensory positioning (PSP) test is a rapid sensory testing method to discriminate the samples by comparing the testing samples to three or more reference samples called poles, and one of the purposes for the development was to determine the taste of water [23,24]. Sixty-five subjects (ages 25–59; 21 males and 44 females) were recruited and voluntarily participated in the test. A 10 min introductory session was conducted for the purpose of this study to outline the PSP method, and the testing procedure prior to the actual testing. Three poles were used as references: poles A, B, and C were composed of TW and deionized water at ratios of 90:10, 50:50, and 10:90, respectively. In the testing booth, the subjects received the three poles, and were asked to taste the pole sample and remember the sensory characteristics of each pole. They received 10 purified water samples one at a time with a Williams Latin Square design [25]. Approximately 30 mL of the sample was placed into a 60 mL transparent plastic cup, and then coded using a three-digit random number. Poles and samples were prepared a day prior to the testing day, and maintained at room temperature (23 °C). For each sample, the subject rated the degree of dissimilarity of the three poles using a 15 cm line scales ranging from “exactly the same” = 0 on the left to “totally different” = 15 on the right, with anchors marked at 1.25 cm from either end. The subjects were allowed to re-taste reference samples at any time (poles A, B, and C), and were then asked to compare the given purified water samples of random-ordered reference samples. The subjects were also asked to comment on the sensory characteristics of the samples in an open-ended question. Reference samples were presented during the test, and allowed the subjects to re-taste samples at any time. The testing was conducted in individualized booths under standard white lighting with a computerized sensory data collection system (Compusense-at-hand, Compusense Inc., Guelph, Canada). Similar sensory characteristics mentioned by each subject were combined after reviewing terms by two professional sensory researchers. Sensory characteristics mentioned by >5% of the subjects were projected onto the result plot as supplementary data in the data analysis.

### 2.5. Consumer Acceptance Test

To compare the acceptability of the purified water samples, 90 subjects (aged 25–59 years; 33 males and 55 females) voluntarily participated in the consumer acceptance test of purified water samples at the Korea Food Research Institute (Wanju-gun, Korea). Purified water samples were bottled a day before the testing date and kept at room temperature. The sample (30 mL) was poured into a 60 mL transparent plastic cup, and coded using three-digit random numbers. The participants evaluated all 10 samples during the one-hour testing session using a 9-point hedonic scale labeled “1 = dislike extremely”, “5 = neither like nor dislike”, and “9 = like extremely”. The test was conducted in individual booths equipped with a computerized data collection system (Compusense-at-hand, Compusense Inc.). The order of sample presentation was determined by a Williams Latin Square design [25]. To prevent sensory fatigue, the participants took a 5 min mandatory rest following evaluation of the fifth sample. After the test, the panelists received financial compensation for their participation.

### 2.6. Statistical Analysis

Analysis of variance (ANOVA), agglomerative hierarchical cluster (AHC) analysis, and principal component analysis (PCA) for physicochemical properties and consumer results were performed using XLSTAT software (Ver. 2017; Addinsoft, Paris, France). Statistical analyses were performed using R version 2.14.1 (R Project for Statistical Computing) and IBM SPSS Statistics for Windows 22 (SPSS Inc., Chicago, IL, USA) for the results from the PSP method. FactoMineR [26] was used to perform multiple factor analysis (MFA) and calculate NRV coefficients. Data from PSP were analyzed using MFA to preserve individual data and compensate for consumer differences when scoring global similarities and differences between samples and poles [24]. MFA was performed by considering the data from each consumer as a separate group of variables. Confidence ellipses were calculated using truncated total bootstrapping, considering the first two dimensions of the configurations [27].

## 3. Results and Discussion

### 3.1. Chemical Analysis of Filtered Water

The results of the chemical analyses of the purified water are presented in Table 1. Purified water through the RO filter showed significant differences in pH and conductivity. The pH was significantly lower than the other samples. Conductivity of PR-RO was the lowest among the samples, since most of minerals were filtered, while PR-RO-PR was the highest, at 129.63 µS/cm. The water purified by PR-RO-PR was stored in a reservoir (Figure 1). During the storage time, some metallic materials of the reservoir leached out to the purified water, and affected the higher conductivity. The mineral and anion contents of the purified water differed with respect to the main filter types. In terms of mineral content, applying PR in the treatment generated a significant decrease in Ca (*p* < 0.05), while the other minerals were not filtered. Among the four main filters, RO was effective in removing minerals from the TW. For UF, CU, and AN, between 2.12 and 2.58 mg/L of Ca was filtered, and the contents were significantly lower than that of TW (*p* < 0.05). Potassium increased after filtration of CU and decreased after filtration of AN (*p* < 0.05). Na and Mg were not filtered through the three filters. In the anion contents, the RO filter removed most of the Cl^−^, NO_3_^−^ and SO_4_^2−^. Less than 0.25 mg/L of Cl^−^ was filtered by the UF, CU, and AN filters. NO_3_^−^ and SO_4_^2−^ were removed in the UF, CU, and AN filters at 0.76–0.83 mg/L, and 0.47–1.82 mg/L, respectively.

The effect of PO after PR and main filter treatment on TW was observed (Table 1). Regarding mineral contents, Ca increased from 0.14 to 0.73 mg/L after PO. The effects of PO on Na, K, and Mg did not show a consistent pattern. Na changed from −0.3 to 0.03 mg/L after PO treatment. K decreased in the UF and CSM (*p* < 0.05) filters, and Mg levels significantly increased in NF and RO (*p* < 0.05). The PO seemed to give water taste for RO treatment by adding Ca and Mg. Regarding anion contents, Cl^-^ was derived from the purification process at the water plant.

NO_3_^−^ and SO_4_^2−^ were generated by heterogeneous and homogeneous reactions of SO_2_ and NO*_x_* emitted from industrial areas [28]. After PR filtration, there was no significant difference in the removal of NO_3_^−^ other than in the PR-RO treatment. In SO_4_^2−^ removal, the main filters were statistically effective (*p* < 0.05), and the RO filter was the most effective in removing all SO_4_^2−^ in the water. Attaching PO decreased NO_3_^−^ and SO_4_^2−^ effectively, while Cl^−^ increased significantly (*p* < 0.05). NO_3_^−^ decreased by 0.18–0.61 mg/L, and SO_4_^2−^ decreased by 0–1.22 mg/L. The Cl^-^ concentration increased by 0.14–0.99 mg/L.

**Figure 2:**
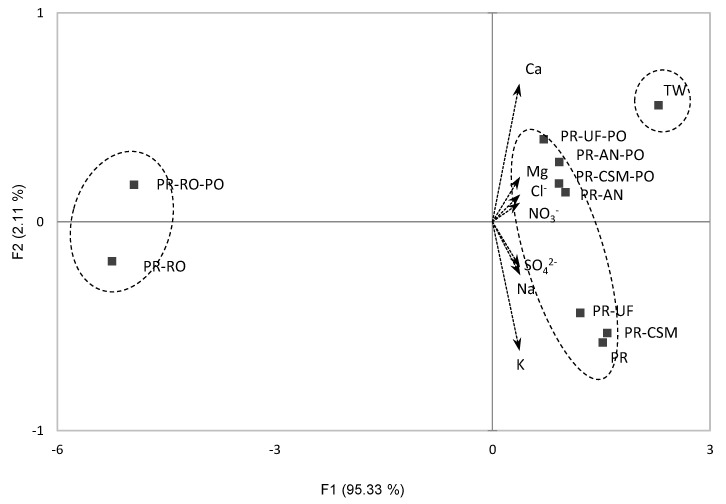
Biplot of the principal component analysis of purified water from mineral and anion contents. Ellipses represents sample clusters from the agglomerative hierarchical clustering analysis. Sample codes refer to Figure 1.

To present overall sample loading to the two-dimensional plot, a PCA was conducted (Figure 2). The PCA plot explained 97.44% of the total variations (F1: 95.33%, F2: 2.11%). Additionally, the samples were grouped into three clusters according to the AHC analysis. The TW was on the right side of the plot, which meant that the TW had the highest contents of minerals and anions. PR with UF, CU, and AN filtered samples were grouped together, and the RO filtered samples were on the left side of the plot, which meant that the RO filtered most of minerals and anions.

### 3.2. Volatile Organic Compounds in Filtered Tap Water

The results for VOCs in filtered tap water using the SPME GC/MSD method are presented in Table 2. A total of 31 VOCs were identified, including 19 hydrocarbons, 8 heterocyclics, 3 acids, and 1 aldehyde. Compared to the previous study, geosmin (trans-1,10-dimethyl-trans-9-decalol) and 2-methyl isoborneol related to malodors in water supply sources were not detected [29]. Fourteen VOCs were detected in TW, namely ethyl caprylate, phenylacrylic acid, hexane, chloroform, bromodichloromethane, dibromochloromethane, 1,2-dimethyl-benzene, 1,4-dimethyl benzene, 1,3-dimethyl-benzene, anethole, estragole, aminobenzimidazole, permetrinic acid methylaminde, and auramine, with the highest total VOC concentrations among the sample at 14.97 µg/Kg. All VOCs in TW were below the TW quality regulations in Korea [30]. Due to the chlorine treatment in municipal water treatment, relatively high concentrations of bromodichloromethane and dibromochloromethane were detected in TW [31]. After the PR filtration, the total VOC content decreased to 4.50 µg/Kg and four VCs, ethyl 6-bromohexanoate, chloroform, anethole, and estragole, were detected. The PR filter removed various VOCs; however, ethyl 6-bromohexanoate, methylcyclopentane, and estragol concentrations increased, which may have been caused by the charcoal in PR. The total concentrations of VOCs after treatment with the UF, CMS, AN, and RO filters were 2.00, 1.70, 3.70, and 2.40 µg/Kg, respectively, and were lower than those after PR treatment.

When the PR filtered water went through the four different main filters (UF, CSM, AN, and RO), VOC contents were 2.00, 1.70, 3.70, and 2.40 µg/Kg, respectively. PR-UF contained hexane, chloroform, and anethole. Chloroform and anethole decreased after PR-CSM treatment, while some VOCs (toluene, cycloheptatriene, and chlorobenzene) were detected that were not detected in the TW and PR samples. Bromodichloromethane concentration after PR-CSM treatment was the highest among the samples, and propenyl vinyl acetylene was newly detected. Hexane, chloroform, anethole, and 1-methyl-2-aminobenzimidazole were present after PR-RO treatment, and were also present in the TW. Further, 3-methyltetrahydrofuran, benzoimide, 6-aminotetralin, and 7-morpholinoindolizidine were detected. The results showed that each main filter could remove different concentrations of VOCs, owing to the different pore sizes, materials, and membrane structures. 

Implemented PO was more effective in removing VOCs. VOC concentrations after PR-UF-PO, PR-CSM-PO, PR-RO-PO, and PR-AN-PO treatments were 0.20, 0.20, 0.20, and 0.50 µg/Kg, respectively. Only hexane was detected after PR-UF-PO, PR-CSM-PO, and PR-AN-PO treatment, while PR-RO-PO contained methylcyclopentane, 4-methyl-1-pentene, cyclohexane, and 2-ethyl-1-butene.

The unpleasant flavor of TW, which is generated during purification at the water plant, is a negative issue in drinking TW [1]. This has caused many consumers to avoid drinking TW directly from the tap, although TW quality is within the permissible limits. POU water treatment significantly influenced the removal of VOCs in tap water. Approximately 70% of VOCs were removed by PR; therefore, simply attaching a PR can remove VOCs significantly. When the main filter was attached after PR treatment, VOCs were reduced by 75.3–88.7%. Even after the addition of PO, >97% of the VOCs were removed. All four main filters effectively removed VOCs, and it is difficult to conclude which was superior.

Although the POU system is this study showed a significant decline in VOCs, this study had some limitations. The TW quality in the Republic of Korea is much better than many countries with soft water, which contains lower concentrations of minerals. Despite demonstrating the effect of removing VOCs, a more drastic filtration effect was not observed as a result of the lower concentration of VOCs in Korean TW. If a lower quality of TW was used in the POU treatment, the ability to remove VOCs and chemicals, and distinguish samples based on sensory characteristics, could be more drastic across filter types.

### 3.3. Discrimination and Consumer Acceptability of Purified Water

**Figure 3:**
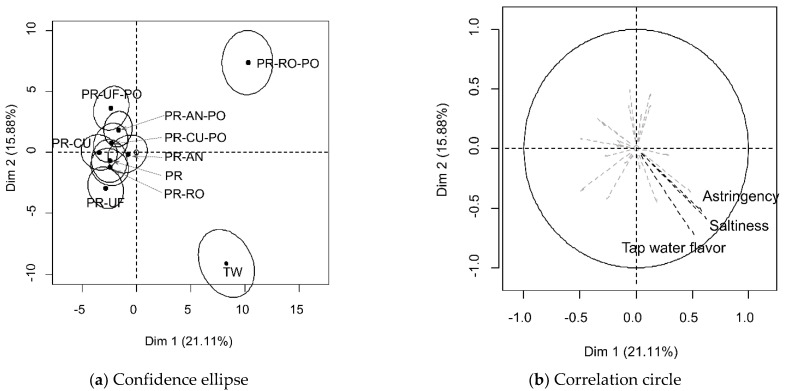
Representation of 10 purified water samples: (**a**) in the first two dimensions of the multiple factor analysis performed on the data from polarized sensory positioning; and (**b**) the corresponding sensory descriptors used as supplementary variables. Sample codes refer to Figure 1.

Sensory quality is one of key factors in accepting and rejecting foods and beverages. Even if a certain food is considered healthy and nutritious, consumers reject tasting the food if it has a bad odor. An “off-flavor” of the water is the main reason for rejecting drinking water [7]. Therefore, appropriate sensory properties of drinking water are key to ensure that people drink water, which is especially relevant since TW contains VOCs resulting from the filtration process. Thus, consumers react to VOCs in TW and determine whether to drink it or not [1,2]. SP methods with open-ended questions regarding the perceived taste of the samples were conducted to investigate how consumers discriminate purified TW in the holistic approach. Discrimination of purified water samples using the PSP method is shown in Figure 3. The purified water samples were divided into three groups (Figure 3a). The TW and PR-RO-PO samples were located at the bottom-right and top-right corners, respectively. The remaining samples were located slightly left in the central area of the plot. The subjects did not discriminate samples clearly, other than the TW and PR-RO-PO samples. Therefore, the effects of the main filter types and PO treatment were limited in the discrimination of purified water, other than the PR-RO-PO. Since the PSP method distinguished the samples according to their holistic characteristics, the reason for the difference cannot be identified [24]. To address this issue, each subject was asked an open-ended question for each sample that was used as supplementary data (Figure 3b). Sensory characteristics with a cos^2^ >0.7 were selected, namely tap water flavor, saltiness, and astringency. The subjects easily identified TW after drinking. The perceived taste difference in the PR-RO-PO samples from the other purified water may be due to the reservoir in the system rather than the VOCs in the sample. The home RO system has a reservoir because the RO requires significant time to purify water due to its low efficacy compared to other filters [32]. The reservoir in this study was connected to the filter using a narrow pipe and tightly sealed to discharge the stored water using the difference in pressure. The metallic inner surface of the reservoir could negatively influence the taste of water. The significantly higher conductivity of PR-RO-PO supported the results of the PSP test (Table 1). The difference between the PR-RO and PR-RO-PO samples was due to the direct collection of the PR-PO sample prior to storage in the reservoir.

**Figure 4:**
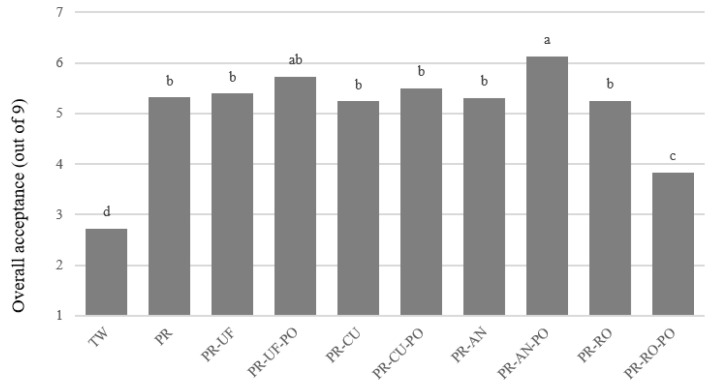
Mean consumer acceptance ratings of purified water samples. Difference letters above bars meant significantly difference at *p* < 0.05 by Tukey’s HSD test. Sample codes refer to Figure 1.

The results of the consumer acceptance test for purified water are shown in Figure 4. The overall acceptance rates of the samples were significantly different across samples (*p* < 0.05). TW had the lowest acceptance, at 2.72 out of 9, and 67.8% of the consumers correctly identified TW from the open-ended question. The higher concentration of VOCs in TW (Table 2) seemed to cause its identification and lower acceptability. Although determining whether the subjects would drink tap water was not part of the pre-questionnaire, they drank filtered water, which has a lower concentration of VOCs during working hours. The off-flavor generated during the filtration process was easily identified in this study. The subjects appeared to recognize that the off-flavor was generated during the filtration process, and its chemical characteristics resulted in the subject rejecting the TW. Water is a bland sample, so minimizing negative characteristics is the key in increasing acceptability, as in a previous study that evaluated cooked rice [33]. PR-AN-PO samples had the highest acceptance rating of 6.13. The other purified samples scored 5.24–5.73 out of 9, and were not significantly different (*p* < 0.05), other than the PR-RO-PO samples. Attaching PO did not influence the acceptance of purified water quality (Table 2). However, the PR-RO-PO sample had the lowest acceptance rating of 3.83 out of 9. The PR-RO-PO sample was also clearly distinguished in the PSP test (Figure 3a), with consumers highlighting notable characteristics in the sample. Of the consumers, 34.4% commented on a medicinal, metallic, or bitter taste for the PR-RO-PO samples, while 11.1% commented on these characteristics for the PR-RO samples in the open-ended questions of the consumer test (results not shown).

The lower acceptance rating of the PR-RO-PO filter may be due to the reservoir in the PR-RO-PO treatment system. The purification efficiency of the RO filter was lower than the other main filters; therefore, the RO purifying system had a reservoir to supply an adequate amount of purified water [32]. When collecting the sample using our RO treatment, the PR-RO was directly collected prior to storage in the reservoir for testing as shown in Figure 1, while the PR-RO-PO filter stored the purified water in the reservoir and was then collected as usual at home. We did not consider the effect of the reservoir during RO treatment. The reservoir was tightly sealed and only connected to the narrow plastic pipe for purified water to move in and out; therefore, we assumed the inside of the reservoir was clean. However, there was reservoir effect reported in the metallic taste of water according to the PSP method and consumer acceptance testing. Hence, a direct comparison between PR-PO and PR-RO-PO may be questionable. However, this issue determined that an appropriate reservoir is necessary for acceptable quality of RO-purified water from an organoleptic perspective.

To closely analyze consumer acceptance, an AHC analysis was conducted (Table 3). The consumers were divided into three clusters according to the level of acceptance: C1 (*n* = 27), C2 (*n* = 47), and C3 (*n* = 16). Consumers in all clusters disliked TWs. As discussed above, only PR-RO-PO samples exhibited a different trend. The results showed that consumers in C2 had a lower acceptance rating for the PR-RO-PO samples than the other clusters, while the remaining purified water samples were rated above 5. Therefore, approximately half of the consumers were sensitive to the taste of purified water and disliked the PR-RO-PO sample.

A comparison of the acceptance of purified water with TW has not been reported recently. Consumers in this study easily identified TW and PR-RO-PO samples, which had distinctive organoleptic characteristics when consumed. Therefore, the off-flavor or off-taste issue influenced the consumer acceptability. The results coincided with the previous survey study on the reasons for drinking bottled water, which found that a majority of consumers drank bottled water because of associated organoleptic issues with TW, rather than safety issues, in Canada and France [1]. Although TW is safe to drink, organoleptic issues were identified as a barrier to drinking TW, which was consistent with previous studies [1,2,3]. Throughout the PSP and consumer acceptance test, consumers did not clearly discriminate filtered water samples other than the PR-RO-PO sample. Even the PR filter was effective at eliminating VOCs (Table 2). By applying POU treatment at home, TW can easily be used as drinking water with lower chemical odors. The PR in this study can filter 1,850 L of TW, which is equivalent to 3700 bottles of water 500 mL in volume. The weight of the PR filter is <1 kg, whereas the weight of 3700 plastic bottles is approximately 55.5 kg. Less than 2% of waste is produced by using the POU system. Further, the price of the PR is less than 1% of the price of 3700 bottles of water. Hence, using POU is more economical and environmentally friendly compared to drinking bottled water.

## 4. Conclusions

POU water treatment removed anions generated during the purification process in water plants (Cl^−^) and derivatives from air pollution (NO_3_^−^ and SO_4_^2−^). The off-flavor in TW generated during the water plant purification process was effectively minimized, although there were some differences in the removal percentages across the filters. POU water treatment also efficiently removed the VOCs that may negatively affect the sensory perception of water as additional filtration steps were applied. Throughout the two sensory evaluations, the organoleptic characteristics of the samples were critical in discriminating and measuring consumer acceptance. TW was clearly differentiated and identified from the filtered water, and was the least accepted among the samples. The effect of the filter in the sensory discrimination was negligible, even when the minimum treatment (PR) was applied. The consumers did not discriminate the purified water samples from the various purification treatments in terms of sensory aspects. Additionally, the difference in acceptance across the purified water was <1, other than the PR-RO-PO sample. These results showed that although there were differences in the ability to remove minerals, anions, and VOCs, the consumers did not identify sensory differences, and showed similar consumer acceptability for the purified water. Further, the PR-RO filter eliminated most minerals and anions; however, the elimination did not influence taste difference significantly. Therefore, the type of filter did not affect the organoleptic issues in the purified water. Simply applying a pre-carbon filter to TW is enough to minimize VOCs, which negatively influence consumer acceptability.

## Figures and Tables

**Table 1:** Results of the chemical analysis of filtered water through various filter systems.

Sample	pH	Conductivity (µS/cm)	Mineral (mg/L)				Anion (mg/L)		
			Ca	Na	K	Mg	Cl^−^	NO_3_^−^	SO_4_^2−^
TW	7.13 ± 0.05 ^bc^	67.97 ± 0.17 ^c^	6.52 ± 0.08 ^a1)^	3.53 ± 0.17 ^abc^	1.05 ± 0.01 ^b^	0.91 ± 0.00 ^a^	7.85 ± 0.00 ^a^	3.64 ± 0.00 ^a^	4.58 ± 0.02 ^a^
PR	7.17 ± 0.01 ^b^	67.10 ± 0.16 ^c^	4.22 ± 0.04 ^cd^	3.75 ± 0.15 ^ab^	1.15 ± 0.02 ^ab^	0.85 ± 0.00 ^a^	7.67 ± 0.02 ^c^	2.79 ± 0.02 ^b^	4.24 ± 0.00 ^b^
PR-UF	7.21 ± 0.01 ^ab^_A_	67.93 ± 0.12 ^c^_B_	3.94 ± 0.15 ^d^_B2)_	3.42 ± 0.23 ^abc^_NS_	1.07 ± 0.08 ^ab^_A_	0.83 ± 0.08 ^a^_NS_	7.68 ± 0.01 ^c^_B_	2.88 ± 0.00 ^b^_A_	3.98 ± 0.00 ^d^_A_
PR-UF-PO	7.06 ± 0.00 ^c^_B_	70.17 ± 0.00 ^b^_A_	4.50 ± 0.00 ^bc^_A_	3.25 ± 0.00 ^c^	0.86 ± 0.00 ^c^_B_	0.89 ± 0.00 ^a^	7.85 ± 0.00 ^a^_A_	2.52 ± 0.00 ^cd^_B_	2.76 ± 0.00 ^h^_B_
PR-CSM	7.27 ± 0.04 ^a^_NS_	68.77 ± 0.41 ^bc^_A_	4.40 ± 0.17 ^bc^_NS3)_	3.86 ± 0.25 ^a^_NS_	1.17 ± 0.05 ^a^_A_	0.87 ± 0.05 ^a^_NS_	7.60 ± 0.01 ^d^_B_	2.81 ± 0.00 ^b^_A_	3.94 ± 0.00 ^e^_A_
PR-CSM-PO	7.21 ± 0.01 ^ab^	67.33 ± 0.26 ^c^_B_	4.54 ± 0.10 ^b^	3.50 ± 0.14 ^abc^	0.91 ± 0.00 ^c^_B_	0.84 ± 0.00 ^a^	7.81 ± 0.00 ^b^_A_	2.63 ± 0.06 ^c^_B_	3.14 ± 0.00 ^fg^_B_
PR-AN	7.16 ± 0.01 ^bc^_NS_	68.37 ± 0.45 ^bc^_NS_	4.37 ± 0.09 ^bc^_B_	3.32 ± 0.01 ^bc^_B_	0.85 ± 0.02 ^c^_NS_	0.82 ± 0.01 ^a^_B_	7.66 ± 0.00 ^c^_B_	2.83 ± 0.00 ^b^_A_	4.11 ± 0.01 ^c^_A_
PR-AN-PO	7.20 ± 0.02 ^ab^	68.83 ± 0.12 ^bc^	4.59 ± 0.04 ^b^_A_	3.35 ± 0.00 ^bc^_A_	0.87 ± 0.02 ^c^	0.89 ± 0.01 ^a^_A_	7.80 ± 0.00 ^b^_A_	2.52 ± 0.08 ^d^_B_	3.43 ± 0.01 ^f^_B_
PR-RO	6.31 ± 0.04 ^e^_B_	56.93 ± 0.25 ^d^_B_	0.00 ± 0.00 ^f^_B_	0.77 ± 0.10 ^d^_NS_	0.21 ± 0.04 ^d^_NS_	0.00 ± 0.00 ^c^_B_	0.32 ± 0.00 ^f^_B_	0.61 ± 0.00 ^e^_A_	0.00 ± 0.00 ^i^_NS_
PR-RO-PO	6.55 ± 0.04 ^d^_A_	129.63 ± 1.76 ^a^_A_	0.73 ± 0.00 ^e^_A_	0.76 ± 0.02 ^d^	0.23 ± 0.00 ^d^	0.19 ± 0.00 ^b^_A_	1.29 ± 0.00 ^e^_A_	0.00 ± 0.00 ^f^_B_	0.00 ± 0.00 ^i^

(1) Different superscripts within a row mean significant differences across the samples by Tukey’s test at *p* < 0.05. (2) Different subscripts within each sample group mean significant differences between samples at *p* < 0.05. (3) NS means not significant between the samples within each group at *p* < 0.05.

**Table 2:** Volatile organic compounds in 11 water samples using GC/MSD.

Compounds	RT (min) ^(1)^	RI ^(1)^	Content (µg/Kg of Water)										
			TW	PR	PR-UF	PR-CU	PR-AN	PR-RO	PR-UF-PO	PR-CU-PO	PR-AN-PO	PR-RO-PO	I.D. ^(1)^
**Acids (3)**													
Ethyl caprylate	20.137	1188	2.40	ND	ND	ND	ND	ND	ND	ND	ND	ND	MS
Ethyl 6-bromohexanoate	20.235	1191	ND	1.00	ND	ND	ND	ND	ND	ND	ND	ND	MS
Phenylacrylic acid	24.005	1329	0.30	ND	ND	ND	ND	ND	ND	ND	ND	ND	MS
**Aldehyde (1)**													
2-Hexenal	5.489	<800	ND	ND	ND	ND	ND	ND	ND	ND	ND	ND	MS/RI
**Hydrocarbons (19)**													
Hexane	3.349	<800	0.10	ND	0.10	ND	0.10	0.10	0.20	0.20	0.20	0.10	MS/RI
Chloroform	3.561	<800	0.07	0.30	0.60	0.30	0.10	0.10	ND	ND	ND	ND	MS
Methylcyclopentane	3.658	<800	ND	ND	ND	ND	ND	ND	ND	ND	ND	0.10	MS
4-Methyl-1-hexene	3.675	<800	ND	ND	ND	ND	ND	ND	ND	ND	ND	ND	MS
3-Methyl-pentane	3.693	<800	ND	ND	ND	ND	ND	ND	ND	ND	ND	ND	MS
4-Methyl-1-pentene	4.059	<800	ND	ND	ND	ND	ND	ND	ND	ND	ND	0.10	MS
Cyclohexane	4.156	<800	ND	ND	ND	ND	ND	ND	ND	ND	ND	0.10	MS
2-Ethyl-1-butene	4.259	<800	ND	ND	ND	ND	ND	ND	ND	ND	ND	0.10	MS
Bromodichloromethane	5.014	<800	3.00	ND	ND	ND	3.30	ND	ND	ND	ND	ND	MS
Toluene	6.617	<800	0.20	ND	ND	0.20	0.10	ND	ND	ND	ND	ND	MS/RI
Propenyl vinyl acetylene	6.702	<800	ND	ND	ND	ND	0.10	ND	ND	ND	ND	ND	MS
Cycloheptatriene	6.851	<800	ND	ND	ND	0.10	ND	ND	ND	ND	ND	ND	MS
Dibromochloromethane	7.469	<800	2.50	ND	ND	ND	ND	ND	ND	ND	ND	ND	MS
Chlorobenzene	9.077	845	ND	ND	ND	0.30	ND	ND	ND	ND	ND	ND	MS
1,2-Dimethyl-benzene	9.901	869	0.10	ND	ND	ND	ND	ND	ND	ND	ND	ND	MS
1,4-Dimethyl-benzene	9.958	870	0.20	ND	ND	ND	ND	ND	ND	ND	ND	ND	MS
1,3-Dimethyl-benzene	10.593	887	0.10	ND	ND	ND	ND	ND	ND	ND	ND	ND	MS
Anethole	21.762	1247	3.20	2.40	1.30	0.50	ND	0.30	ND	ND	ND	ND	MS
Estragole	22.964	1289	0.50	0.80	ND	0.30	ND	ND	ND	ND	ND	ND	MS/RI
**Heterocyclics (8)**													
3-Methyltetrahydrofuran	3.372	<800	ND	ND	ND	ND	ND	0.10	ND	ND	ND	ND	MS
Benzoimide	4.516	<800	ND	ND	ND	ND	ND	0.30	ND	ND	ND	ND	MS
6-Aminotetralin	4.602	<800	ND	ND	ND	ND	ND	0.30	ND	ND	ND	ND	MS
1-Methyl-2-aminobenzimidazole	4.642	<800	0.30	ND	ND	ND	ND	0.30	ND	ND	ND	ND	MS
Permetrinic acid methylaminde	5.272	<800	2.00	ND	ND	ND	ND	ND	ND	ND	ND	ND	MS
Benzenehexanamine	6.542	<800	ND	ND	ND	ND	ND	ND	ND	ND	ND	ND	MS
7-Morpholinoindolizidine	7.99	810	ND	ND	ND	ND	ND	0.90	ND	ND	ND	ND	MS
Auramine	17.705	1105	1.60	ND	ND	ND	ND	ND	ND	ND	ND	ND	MS
Total content			14.97	4.50	2.00	1.70	3.70	2.40	0.20	0.20	0.20	0.50	

(1) RT, RI, and I.D. mean retention time, retention index, and identification, respectively.

**Table 3:** Mean consumer acceptance ratings of purified water samples for each cluster generated by the agglomerative hierarchical cluster analysis. Sample codes refer to Figure 1.

Sample	C1 (*n* = 27)	C2 (*n* = 47)	C3 (*n* = 16)
TW	3.52 ^b^	1.91 ^b^	3.75 ^b^
PR	5.07 ^a^	5.26 ^a^	5.94 ^a^
PR-UF	4.74 ^ab^	5.28 ^a^	6.56 ^a^
PR-CU	4.30 ^ab^	5.28 ^a^	6.75 ^a^
PR-AN	4.59 ^ab^	5.38 ^a^	6.31 ^a^
PR-RO	4.87 ^a^	5.11 ^a^	6.31 ^a^
PR-UF-PO	5.37 ^a^	5.70 ^a^	6.44 ^a^
PR-CU-PO	5.48 ^a^	5.28 ^a^	6.19 ^a^
PR-AN-PO	5.48 ^a^	6.13 ^a^	7.25 ^a^
PR-RO-PO	5.41 ^a^	2.09 ^b^	6.31 ^a^

(1) Different superscripts within a row mean significant differences across the samples by Tukey’s HSD test at *p* < 0.05.

## Data Availability

The data presented in this study are available upon request from the corresponding author. The data can be offered to the proper persons with an objective goal for the use of the data.
